# Hydrogen-bonding patterns in 2,2-bis­(4-methyl­phen­yl)hexa­fluoro­propane pyridinium and ethyl­enedi­ammonium salt crystals

**DOI:** 10.1107/S2056989020005575

**Published:** 2020-04-24

**Authors:** Haruki Sugiyama

**Affiliations:** a Research and Education Center for Natural Sciences, Keio University, Hiyoshi, 4-1-1, Kohoku, Yokohama, Japan

**Keywords:** crystal structure, organic salt, hydrogen-bonding networks

## Abstract

The crystal structures of two salt crystals of 2,2-bis­(4-methyl­phen­yl)hexa­fluoro­propane (Bmphfp) with amines, namely, dipyridinium 4,4′-(1,1,1,3,3,3-hexa­fluoro­propane-2,2-di­yl)dibenzoate 4,4′-(1,1,1,3,3,3-hexa­fluoro­propane-2,2-di­yl)di­benzoic acid, 2C_5_H_6_N^+^·C_17_H_8_F_6_O_4_^2−^·C_17_H_10_F_6_O_4_, (**1**), and a monohydrated ethyl­enedi­ammonium salt ethane-1,2-diaminium 4,4′-(1,1,1,3,3,3-hexa­fluoro­propane-2,2-di­yl)dibenzoate monohydrate, C_2_H_10_N_2_^2+^·C_17_H_8_F_6_O_4_^2−^·H_2_O, (**2**), are reported. Compounds **1** and **2** crystallize, respectively, in space group *P*2_1_/*c* with *Z*′ = 2 and in space group *Pbca* with *Z*′ = 1. The crystals of compound **1** contain neutral and anionic Bmphfp mol­ecules, and form a one-dimensional hydrogen-bonded chain motif. The crystals of compound **2** contain anionic Bmphfp mol­ecules, which form a complex three-dimensional hydrogen-bonded network with the ethyl­enedi­amine and water mol­ecules.

## Abstract 2

The crystal structures of two salt crystals of 2,2-bis­(4-methyl­phen­yl)hexa­fluoro­propane (Bmphfp) with amines, namely, dipyridinium 4,4′-(1,1,1,3,3,3-hexa­fluoro­propane-2,2-di­yl)dibenzoate 4,4′-(1,1,1,3,3,3-hexa­fluoro­propane-2,2-di­yl)di­benzoic acid (**1**) and a monohydrated ethyl­enedi­ammonium salt ethane-1,2-diaminium 4,4′-(1,1,1,3,3,3-hexa­fluoro­propane-2,2-di­yl)dibenzoate monohydrate (**2**) are reported.

## Chemical context   

In recent years, porous organic frameworks have been researched extensively because of their structural versatility and potential applications in gas storage and separation and as catalysts and chemical sensors (He *et al.*, 2011 ▸). Hydrogen-bonded organic frameworks (HOFs), which are constructed *via* inter­molecular hydrogen bonds, are being actively investigated for such applications (Hisaki *et al.*, 2018 ▸). HOFs are basically flexible to allow solution-based fabrication/reassembly and dynamic structural conversion as compared to covalent organic frameworks (COFs) (Miyano *et al.*, 2016 ▸). Several multiple-carb­oxy­lic acids are reported to create HOFs *via* carb­oxy­lic dimers (Bassanetti *et al.*, 2016 ▸; Hisaki, 2020 ▸). 2,2-Bis(4-methyl­phen­yl)hexa­fluoro­propane (Bmphfp) is a V-shaped di-carb­oxy­lic acid forming a one-dimensional hydrogen-bonded chain in the crystal structure (Tang *et al.*, 2010 ▸). The HOFs based on carb­oxy­lic acids can be modified or rebuilt by salt formation with various organic bases (Galcera *et al.*, 2012 ▸).
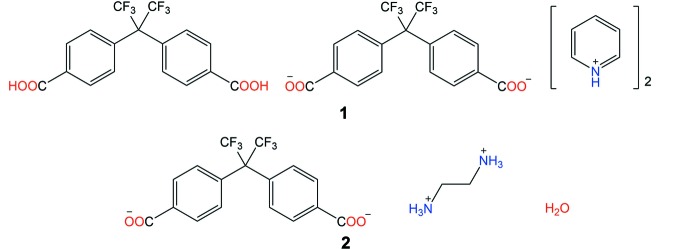


The crystal structures of Bmphfp pyridinium (**1**) and ethyl­enedi­ammonium (**2**) salts are reported herein with a focus on the differences in the hydrogen-bonding patterns.

## Structural commentary   

**Figure 1:**
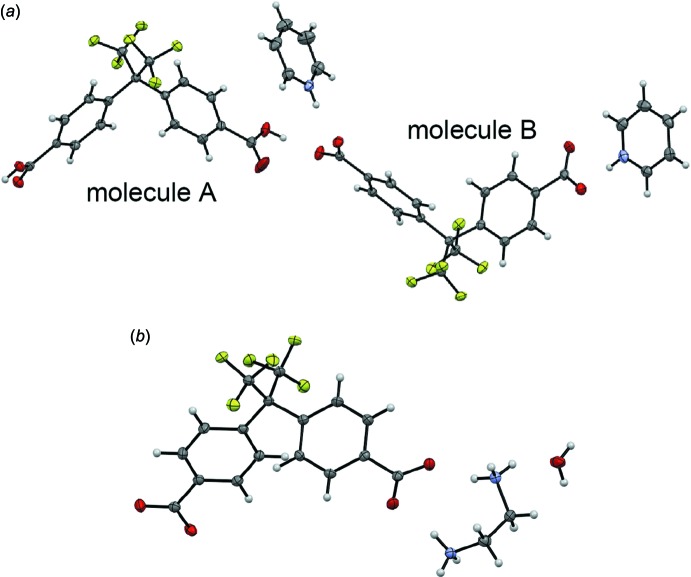
Mol­ecular structures of (*a*) compound **1**, and (*b*) compound **2**. Displacement ellipsoids are drawn at the 50% probability level.

Compound **1** crystallizes in the monoclinic space group *P*2_1_/*c* with two Bmphfp mol­ecules and two pyridinium cations in the asymmetric unit (Fig. 1 ▸ *a*). Inter­estingly, one of the two Bmphfp mol­ecules is neutral and the other is anionic. The C—O bond lengths of the carb­oxy groups are summarized in Table 1 ▸, which suggests that mol­ecules *A* and *B* are in the neutral and divalent anionic forms, respectively. The two benzene rings are twisted with respect to each other, forming dihedral angles of 72.19 (6) and 69.98 (6)°. On the other hand, compound **2** crystallizes in the ortho­rhom­bic space group *Pbcn*. The asymmetric unit comprises one Bmphfp anion, one ethyl­enedi­ammonium cation and one water mol­ecule (Fig. 1 ▸ *b*). The C—O bond lengths shown in Table 1 ▸ confirm that the Bmphfp mol­ecule is in the divalent anionic form. The benzene rings are twisted with a dihedral angle of 64.47 (6)°. The N—C—C—N torsion angle in the ethyl­enedi­ammonium cation is 72.72 (14)°.

## Supra­molecular features   

**Figure 2:**
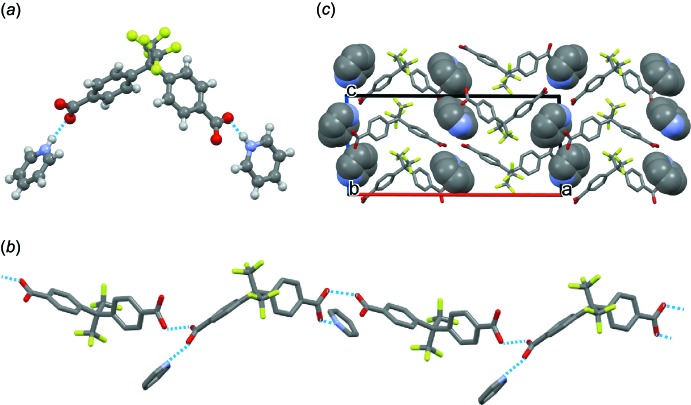
Crystal structure of compound **1**. (*a*) Hydrogen bonds between the Bmphfp and pyridine mol­ecules. (*b*) hydrogen-bonded chain of Bmphfp mol­ecules. (*c*) Mol­ecular packing along the *b*-axis direction.

In the crystal of compound **1**, the pyridine mol­ecules form strong N—H⋯O hydrogen bonds with the carboxyl groups of Bmphfp mol­ecule *B* (Table 2 ▸, Fig. 2 ▸ *a*). The neutral (*A*) and anionic (*B*) Bmphfp mol­ecules form a one-dimensional hydrogen-bonded chain motif along the *a*-axis direction (Fig. 2 ▸ *b*). The lengths of the negative charge-assisted O—H⋯O hydrogen bonds, 2.5732 (13) and 2.5125 (13) Å, are shorter than in the common carboxyl dimer [2.643 Å; the mean value calculated from 505 research hits in the Cambridge Structural Database (CSD version 5.41, November 2019 update; Groom *et al.*, 2016 ▸)]. Fig. 2 ▸ *c* shows the crystal packing of compound **1**. The pyridine mol­ecules are located between the hydrogen-bonded chains of Bmphfp mol­ecules. There are weak C—H⋯F and F⋯F inter­actions between the Bmphfp and pyridine mol­ecules or between Bmphfp mol­ecules. The shortest inter­atomic distances are 3.159 (1) Å (C⋯F) and 2.696 (1) Å (F⋯F), respectively.

**Figure 3:**
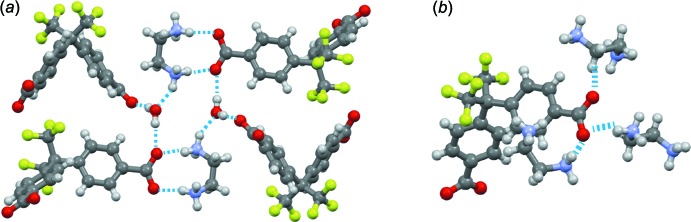
Crystal structure of compound **2**. (*a*) Hydrogen bonds between Bmphfp, ethyl­enedi­amine, and water mol­ecules. (*b*) Hydrogen bonds between Bmphfp and ethyl­enedi­amine mol­ecules.

In the crystal of compound **2**, one carb­oxy­lic group of the Bmphfp mol­ecule is linked to an ethyl­enedi­ammonium cation by two N—H⋯O hydrogen bonds. The N⋯O inter­atomic distances are 2.7749 (14) and 2.8015 (14) Å, respectively (Table 3 ▸). The other carb­oxy­lic group forms N—H⋯O hydrogen bonds with three surrounding ethyl­enedi­ammonium cations (Fig. 3 ▸ *a*). Therefore, five of the six hydrogen-atom donors of the ethyl­enedi­ammonium cations are connected to Bmphfp mol­ecules, resulting in a complex three-dimensional hydrogen-bonding network. The water mol­ecule is linked to both Bmphfp and ethyl­enedi­amine mol­ecules *via* two O—H⋯O and one N—H⋯O hydrogen bonds. Thus, the water mol­ecules are highly stabilized by these inter­molecular inter­actions in the crystal structure (Fig. 3 ▸ *b*). Weak C—H⋯F and F⋯F inter­actions are observed between Bmphfp mol­ecules, resulting inter­atomic distances of 3.493 (1) Å (C⋯F) and 2.890 (1) Å (F⋯F), respectively. In compound **2**, the Bmphfp mol­ecules do not form a discrete 1-D hydrogen bond chain motif as observed in compound **1** because the one carboxyl group is terminated by an ethyl­enedi­amine mol­ecule.

## Hirshfeld surface analysis   

**Figure 4:**
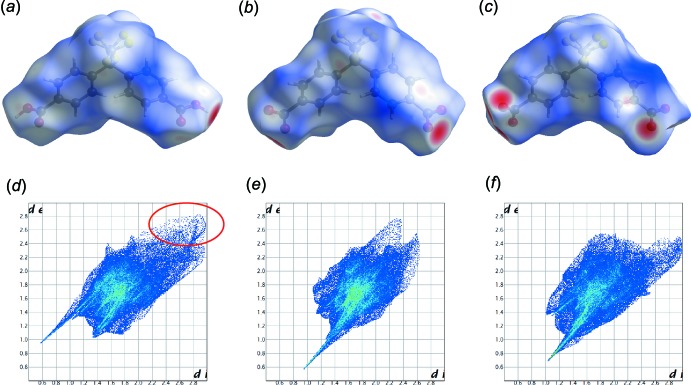
Hirshfeld surface mapped over *d*_norm_ of (*a*) Bmphfp mol­ecule *A* and (*b*) Bmphfp mol­ecule *B* in compound **1**, and (*c*) the Bmphfp mol­ecule in compound **2**. Two-dimensional fingerprint plots of these Bmphfp mol­ecules are shown in (*d*)–(*f*), respectively.

Hirshfeld surfaces (McKinnon *et al.*, 2007 ▸) and their associated two-dimensional fingerprint plots (Spackman & McKinnon, 2002 ▸) were calculated using *CrystalExplorer17* (Turner *et al.*, 2017 ▸). The *d*_norm_ surface of the Bmphfp mol­ecules in compounds **1** and **2** are shown in Fig. 4 ▸ *a*–*c*. The red colour highlights the surface areas where there are strong inter­actions such as O—H⋯O hydrogen bonds. In compound **1**, there are two independent Bmphfp mol­ecules, *A* and *B*. There is no significant difference in the contact contributions of each of the mol­ecules (Tables 4 ▸ and 5 ▸). However, in the fingerprint plots, mol­ecule *B* has no contribution from contacts with a long inter­atomic distance (highlighted by the red circle in Fig. 4 ▸ *a*) compared to mol­ecule *A*. Thus, mol­ecule *B* is more closely packed with the surrounding mol­ecules in the crystal than mol­ecule *A*. This may be due to the difference in the ionic state between neutral mol­ecule *A* and anionic mol­ecule *B*. Compound **1** (mol­ecule *A* and *B*) has strong hydrogen-bonding inter­actions, with similar but slightly weaker inter­actions for compound **2**. The contributions to the Hirshfeld surface for **2** are listed in Table 6 ▸.

## Database survey   

A search of the Cambridge Structural Database (CSD version 5.41, November 2019 update; Groom *et al.*, 2016 ▸) for crystal structures with 2,2-bis­(4-methyl­phen­yl)hexa­fluoro­propane gave 244 hits. Two polymorphs of Bmphfp, TUPNOI (Tang *et al.*, 2010 ▸) and TUPNOI01 (Pachfule *et al.*, 2010 ▸), have been reported. In both crystal structures, the Bmphfp mol­ecules form similar one-dimensional hydrogen-bonding motifs *via* carb­oxy­lic dimers. However, there is only one structure of a Bmphfp organic salt with 1,4-bis­(2-pyridyl­amino­meth­yl)benzene (EFOLIW; Tripuramallu, 2014 ▸). Other results are inorganic salts or metal complex salts (WAQTUF; Platero-Prats *et al.*, 2012 ▸) including metal–organic frameworks (KUXRAX; Prats *et al.*, 2010 ▸; Platero-Prats *et al.*, 2010 ▸; AVILAT; Wang *et al.*, 2011 ▸).

## Synthesis and crystallization   

The reagents 2,2-bis­(4-methyl­phen­yl)hexa­fluoro­propane, pyridine and ethyl­enedi­amine were purchased from TCI Co., Ltd. (Japan). 2,2-Bis(4-methyl­phen­yl)hexa­fluoro­propane (2.5 mmol, 0.083 g) was dissolved in methanol 10 mL. The Bmphfp solution was mixed into 5 mL of a 1.0 *M* pyridine methanol solution under stirring. After slow evaporation, colourless plate-like crystals of compound **1** were obtained. When the Bmphfp solution was mixed into 5 ml of a 1.0 *M* ethyl­enedi­amine methanol solution under stirring, colourless needle-like crystals of compound **2** were obtained.

## Refinement   

Crystal data, data collection and structure refinement details are summarized in Table 7 ▸. H atoms were positioned geometrically and refined using a riding model: C—H = 0.93, O—H = 0.82, N*sp* ^2^—H = 0.86, N*sp* ^3^—H = 0.89 Å with *U*_iso_(H) = 1.2*U*_eq_(C, N,O).

## Supplementary Material

Crystal structure: contains datablock(s) 1, 2, global. DOI: 10.1107/S2056989020005575/lh5959sup1.cif

Structure factors: contains datablock(s) 1. DOI: 10.1107/S2056989020005575/lh59591sup2.hkl

Structure factors: contains datablock(s) 2. DOI: 10.1107/S2056989020005575/lh59592sup3.hkl

CCDC references: 1998170, 1998169

Additional supporting information:  crystallographic information; 3D view; checkCIF report

## Figures and Tables

**Table 1:** Bond lengths (Å) in the carboxyl groups of compounds **1** and **2**

	Bond	length	bond	length
Compound **1**	C2—O2	1.215 (2)	C2—O3	1.321 (2)
mol­ecule *A*	C25—O26	1.217 (2)	C25—O27	1.307 (2)
Compound **1**	C29—O28	1.260 (2)	C29—O30	1.260 (2)
mol­ecule *B*	C54—O53	1.251 (2)	C54—O55	1.268 (2)
Compound **2**	C2—O1	1.252 (2)	C2—O3	1.265 (2)
	C25—O26	1.258 (2)	C25—O27	1.261 (2)

**Table 2:** Hydrogen-bond geometry (Å, °) for **1** [Chem scheme1]

*D*—H⋯*A*	*D*—H	H⋯*A*	*D*⋯*A*	*D*—H⋯*A*
O3—H3⋯O55^i^	0.84	1.75	2.5732 (13)	164
O27—H27⋯O28	0.84	1.68	2.5125 (13)	172
N56—H56⋯O30	0.88	1.79	2.6488 (15)	165
N62—H62⋯O53	0.88	1.67	2.5472 (15)	177

Symmetry code: (i) 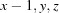.

**Table 3:** Hydrogen-bond geometry (Å, °) for **2** [Chem scheme1]

*D*—H⋯*A*	*D*—H	H⋯*A*	*D*⋯*A*	*D*—H⋯*A*
O32—H32*A*⋯O27^i^	0.85	1.92	2.7656 (14)	175
O32—H32*B*⋯O26^ii^	0.85	1.93	2.7731 (13)	170
N28—H28*A*⋯O1^iii^	0.91	1.87	2.7749 (14)	171
N28—H28*B*⋯O27	0.91	1.87	2.7609 (14)	165
N28—H28*C*⋯O3^iv^	0.91	2.00	2.8015 (14)	146
N31—H31*A*⋯O26	0.91	1.82	2.6933 (14)	160
N31—H31*B*⋯O32	0.91	1.91	2.7288 (14)	149
N31—H31*C*⋯O3^v^	0.91	1.91	2.7220 (14)	148

Symmetry codes: (i) 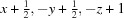; (ii) 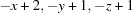; (iii) 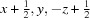; (iv) 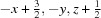; (v) 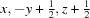.

**Table 4:** Percentage contributions to the Hirshfeld surface of the Bmphfp mol­ecule *A* in compound **1**

OutsideInside	F	O	H	N	O	Total
C	3.5	0.5	7.9	0.4	1.3	13.6
F	6.0	0.7	16.5		3.6	26.9
H	12.0	8.2	18.7	0.3	2.5	41.8
O	0.3	0.8	14.8	0.5	1.3	17.7
Total	21.9	10.3	57.8	1.3	8.8	

**Table 5:** Percentage contributions to the Hirshfeld surface of the Bmphfp mol­ecule *B* in compound **1**

OutsideInside	F	O	H	N	O	Total
C	4.9	0.1	5.0	0.9	3.9	14.9
F	6.1		17.1		3.4	26.3
H	12.0	2.3	17.1	0.2	3.5	35.0
O	0.6	0.7	21.4	0.1	0.7	23.6
Total	23.5	3.2	60.7	1.2	11.4	

**Table 6:** Percentage contributions to the Hirshfeld surface of the Bmphfp mol­ecule in compound **2**

OutsideInside	F	O	H	O	Total
C	2.9	0.7	10.5	0	14.2
F	?6.9	0.6	16.9	2.5	27.0
H	11.7	2.3	17.6	2.5	34.1
O	0.2	0.6	23.6	0.3	24.7
Total	?21.8	4.3	68.6	5.3	

**Table 7:** Experimental details

	**1**	**2**
Crystal data		
Chemical formula	2C_5_H_6_N^+^·C_17_H_8_F_6_O_4_^2−^·C_17_H_10_F_6_O_4_	C_2_H_10_N_2_^2+^·C_17_H_8_F_6_O_4_^2−^·H_2_O
*M*_r_	471.35	470.37
Crystal system, space group	Monoclinic, *P*2_1_/*c*	Orthorhombic, *P* *b* *c* *a*
Temperature (K)	93	93
*a*, *b*, *c* (Å)	25.5453 (7), 13.4125 (4), 11.8879 (4)	13.2518 (3), 12.1773 (3), 25.8419 (6)
α, β, γ (°)	90, 91.644 (3), 90	90, 90, 90
*V* (Å^3^)	4071.4 (2)	4170.14 (17)
*Z*	4	8
Radiation type	Cu *K*α	Cu *K*α
μ (mm^−1^)	1.25	1.26
Crystal size (mm)	0.3 × 0.25 × 0.05	0.22 × 0.1 × 0.05

Data collection		
Diffractometer	XtaLAB Synergy R, DW system, HyPix	XtaLAB Synergy R, DW system, HyPix
Absorption correction	Multi-scan (*CrysAlis PRO*; Rigaku OD, 2019 ▸)	Multi-scan (*CrysAlis PRO*; Rigaku OD, 2019 ▸)
*T*_min_, *T*_max_	0.743, 0.940	0.855, 0.940
No. of measured, independent and observed [*I* > 2σ(*I*)] reflections	30654, 8170, 7361	15896, 4198, 3809
*R*_int_	0.031	0.029
(sin θ/λ)_max_ (Å^−1^)	0.630	0.630

Refinement		
*R*[*F* ^2^ > 2σ(*F* ^2^)], *wR*(*F* ^2^), *S*	0.033, 0.087, 1.03	0.032, 0.082, 1.04
No. of reflections	8170	4198
No. of parameters	597	294
H-atom treatment	H-atom parameters constrained	H-atom parameters constrained
Δρ_max_, Δρ_min_ (e Å^−3^)	0.71, −0.47	0.29, −0.24

Computer programs: *CrysAlis PRO* (Rigaku OD, 2019 ▸), *SHELXT2018/2* (Sheldrick, 2015*a* ▸), *SHELXL2018/3* (Sheldrick, 2015*b* ▸) and *OLEX2* (Dolomanov *et al.*, 2009 ▸).

## supplementary crystallographic information

### Dipyridinium 4,4'-(1,1,1,3,3,3-hexafluoropropane-2,2-diyl)dibenzoate 4,4'-(1,1,1,3,3,3-hexafluoropropane-2,2-diyl)dibenzoic acid (1) Crystal data

**Table:**

2C_5_H_6_N^+^·C_17_H_8_F_6_O_4_^2^^−^·C_17_H_10_F_6_O_4_	*F*(000) = 1920
*M**_r_* = 471.35	*D*_x_ = 1.538 Mg m^−^^3^
Monoclinic, *P*2_1_/*c*	Cu *K*α radiation, λ = 1.54184 Å
*a* = 25.5453 (7) Å	Cell parameters from 18343 reflections
*b* = 13.4125 (4) Å	θ = 3.4–76.1°
*c* = 11.8879 (4) Å	µ = 1.25 mm^−^^1^
β = 91.644 (3)°	*T* = 93 K
*V* = 4071.4 (2) Å^3^	Plate, colourless
*Z* = 4	0.3 × 0.25 × 0.05 mm

### Dipyridinium 4,4'-(1,1,1,3,3,3-hexafluoropropane-2,2-diyl)dibenzoate 4,4'-(1,1,1,3,3,3-hexafluoropropane-2,2-diyl)dibenzoic acid (1) Data collection

**Table:**

XtaLAB Synergy R, DW system, HyPix diffractometer	8170 independent reflections
Radiation source: Rotating-anode X-ray tube, Rigaku (Cu) X-ray Source	7361 reflections with *I* > 2σ(*I*)
Mirror monochromator	*R*_int_ = 0.031
Detector resolution: 10.0000 pixels mm^-1^	θ_max_ = 76.3°, θ_min_ = 3.5°
ω scans	*h* = −30→32
Absorption correction: multi-scan (CrysAlisPro; Rigaku OD, 2019)	*k* = −16→13
*T*_min_ = 0.743, *T*_max_ = 0.940	*l* = −13→14
30654 measured reflections	

### Dipyridinium 4,4'-(1,1,1,3,3,3-hexafluoropropane-2,2-diyl)dibenzoate 4,4'-(1,1,1,3,3,3-hexafluoropropane-2,2-diyl)dibenzoic acid (1) Refinement

**Table:**

Refinement on *F*^2^	Primary atom site location: dual
Least-squares matrix: full	Hydrogen site location: inferred from neighbouring sites
*R*[*F*^2^ > 2σ(*F*^2^)] = 0.033	H-atom parameters constrained
*wR*(*F*^2^) = 0.087	*w* = 1/[σ^2^(*F*_o_^2^) + (0.0395*P*)^2^ + 1.9293*P*] where *P* = (*F*_o_^2^ + 2*F*_c_^2^)/3
*S* = 1.03	(Δ/σ)_max_ = 0.001
8170 reflections	Δρ_max_ = 0.71 e Å^−^^3^
597 parameters	Δρ_min_ = −0.47 e Å^−^^3^
0 restraints	

### Dipyridinium 4,4'-(1,1,1,3,3,3-hexafluoropropane-2,2-diyl)dibenzoate 4,4'-(1,1,1,3,3,3-hexafluoropropane-2,2-diyl)dibenzoic acid (1) Special details

**Table:**

Geometry. All esds (except the esd in the dihedral angle between two l.s. planes) are estimated using the full covariance matrix. The cell esds are taken into account individually in the estimation of esds in distances, angles and torsion angles; correlations between esds in cell parameters are only used when they are defined by crystal symmetry. An approximate (isotropic) treatment of cell esds is used for estimating esds involving l.s. planes.

### Dipyridinium 4,4'-(1,1,1,3,3,3-hexafluoropropane-2,2-diyl)dibenzoate 4,4'-(1,1,1,3,3,3-hexafluoropropane-2,2-diyl)dibenzoic acid (1) Fractional atomic coordinates and isotropic or equivalent isotropic displacement parameters (Å^2^)

**Table:**

	*x*	*y*	*z*	*U*_iso_*/*U*_eq_	
F12	0.22833 (3)	1.08247 (6)	0.68130 (7)	0.02200 (17)	
F17	0.22585 (3)	0.91369 (6)	0.96846 (6)	0.02322 (17)	
F40	0.70683 (3)	0.41820 (6)	0.27299 (7)	0.02356 (17)	
F44	0.76965 (3)	0.72779 (6)	0.13841 (7)	0.02456 (18)	
F13	0.26623 (3)	1.08382 (6)	0.84616 (7)	0.02303 (17)	
F16	0.30597 (3)	0.91053 (6)	0.91890 (7)	0.02226 (17)	
F18	0.25655 (3)	0.78308 (6)	0.88772 (7)	0.02241 (17)	
F41	0.74214 (3)	0.42826 (6)	0.11122 (7)	0.02445 (18)	
F46	0.79621 (3)	0.58962 (6)	0.06906 (7)	0.02426 (18)	
F42	0.18370 (3)	1.05339 (6)	0.82787 (7)	0.02393 (18)	
F45	0.71737 (3)	0.63923 (7)	0.03330 (7)	0.02663 (19)	
F39	0.66577 (3)	0.49010 (6)	0.13508 (7)	0.02771 (19)	
O30	0.56288 (4)	0.74265 (8)	0.56887 (8)	0.0231 (2)	
O3	0.02557 (4)	0.69282 (7)	0.63890 (8)	0.0213 (2)	
H3	−0.001290	0.669702	0.605883	0.032*	
O1	0.03424 (4)	0.77646 (7)	0.47641 (8)	0.0240 (2)	
O27	0.45867 (4)	0.89389 (8)	0.49908 (9)	0.0266 (2)	
H27	0.486853	0.870972	0.475153	0.040*	
O53	0.93843 (4)	0.45833 (8)	0.54257 (9)	0.0265 (2)	
O28	0.54442 (4)	0.84034 (8)	0.42017 (9)	0.0252 (2)	
O55	0.93586 (4)	0.62306 (8)	0.57396 (9)	0.0272 (2)	
N56	0.52505 (5)	0.87381 (9)	0.71195 (10)	0.0224 (2)	
H56	0.533196	0.823332	0.668526	0.027*	
N62	1.00463 (5)	0.43556 (9)	0.70485 (10)	0.0232 (2)	
H62	0.982590	0.444347	0.647461	0.028*	
O26	0.43599 (5)	0.73301 (10)	0.50208 (16)	0.0605 (5)	
C47	0.78662 (5)	0.56715 (10)	0.29798 (11)	0.0162 (2)	
C31	0.61372 (5)	0.72459 (10)	0.40592 (11)	0.0163 (2)	
C7	0.19098 (5)	0.87600 (9)	0.72089 (11)	0.0163 (2)	
C52	0.80108 (5)	0.64725 (10)	0.36757 (11)	0.0186 (3)	
H52	0.780770	0.706591	0.365868	0.022*	
C32	0.62560 (5)	0.75871 (10)	0.29904 (11)	0.0169 (2)	
H32	0.606557	0.813212	0.267331	0.020*	
C19	0.28904 (5)	0.90407 (10)	0.69532 (11)	0.0162 (2)	
C29	0.57024 (5)	0.77278 (10)	0.47017 (11)	0.0180 (3)	
C36	0.64238 (5)	0.64523 (10)	0.45168 (11)	0.0182 (3)	
H36	0.634564	0.620820	0.524315	0.022*	
C33	0.66487 (5)	0.71425 (9)	0.23808 (11)	0.0168 (2)	
H33	0.671986	0.737681	0.164671	0.020*	
C4	0.09858 (5)	0.79697 (9)	0.62440 (11)	0.0173 (3)	
C35	0.68223 (5)	0.60161 (10)	0.39192 (11)	0.0178 (3)	
H35	0.701822	0.548206	0.424533	0.021*	
C34	0.69390 (5)	0.63546 (9)	0.28405 (11)	0.0157 (2)	
C50	0.87447 (5)	0.55365 (10)	0.44332 (11)	0.0181 (3)	
C48	0.81786 (5)	0.48191 (10)	0.29880 (11)	0.0184 (3)	
H48	0.809559	0.428344	0.249142	0.022*	
C49	0.86115 (5)	0.47505 (10)	0.37207 (11)	0.0186 (3)	
H49	0.881768	0.416092	0.373382	0.022*	
C8	0.15374 (5)	0.83113 (10)	0.78814 (11)	0.0188 (3)	
H8	0.159499	0.828271	0.867374	0.023*	
C51	0.84482 (5)	0.64073 (10)	0.43901 (11)	0.0191 (3)	
H51	0.854579	0.695867	0.485179	0.023*	
C2	0.04955 (5)	0.75471 (10)	0.57097 (11)	0.0186 (3)	
C24	0.32764 (5)	0.97498 (10)	0.67589 (11)	0.0185 (3)	
H24	0.323435	1.041261	0.702336	0.022*	
C37	0.73718 (5)	0.57962 (9)	0.22137 (11)	0.0163 (2)	
C11	0.22998 (5)	1.03694 (10)	0.78160 (11)	0.0185 (3)	
C9	0.10838 (5)	0.79069 (10)	0.74009 (11)	0.0195 (3)	
H9	0.083840	0.758493	0.786396	0.023*	
C5	0.13488 (5)	0.84415 (10)	0.55735 (11)	0.0192 (3)	
H5	0.128171	0.850060	0.478619	0.023*	
C22	0.37889 (5)	0.85221 (11)	0.58076 (11)	0.0200 (3)	
C20	0.29548 (5)	0.80725 (10)	0.65561 (11)	0.0196 (3)	
H20	0.269000	0.758830	0.666859	0.023*	
C6	0.18083 (5)	0.88259 (10)	0.60500 (11)	0.0185 (3)	
H6	0.205628	0.913677	0.558374	0.022*	
C38	0.71313 (5)	0.47819 (10)	0.18464 (11)	0.0196 (3)	
C54	0.92002 (5)	0.54511 (11)	0.52634 (11)	0.0203 (3)	
C15	0.25742 (5)	0.88267 (10)	0.88711 (11)	0.0182 (3)	
C10	0.24143 (5)	0.92433 (9)	0.76917 (11)	0.0162 (3)	
C23	0.37220 (5)	0.94944 (10)	0.61817 (11)	0.0196 (3)	
H23	0.398102	0.998398	0.604258	0.023*	
C67	1.03435 (5)	0.35341 (11)	0.70980 (12)	0.0231 (3)	
H67	1.031301	0.305248	0.651379	0.028*	
C21	0.34043 (5)	0.78123 (10)	0.59972 (12)	0.0219 (3)	
H21	0.344950	0.714718	0.574307	0.026*	
C43	0.75479 (5)	0.63450 (10)	0.11453 (11)	0.0198 (3)	
C66	1.06937 (6)	0.33770 (11)	0.79865 (13)	0.0258 (3)	
H66	1.090530	0.279470	0.801645	0.031*	
C25	0.42714 (5)	0.82031 (12)	0.52194 (13)	0.0256 (3)	
C63	1.00803 (6)	0.50452 (11)	0.78613 (13)	0.0273 (3)	
H63	0.986587	0.562295	0.780960	0.033*	
C61	0.48846 (6)	0.86140 (12)	0.78893 (13)	0.0280 (3)	
H61	0.472128	0.798293	0.797199	0.034*	
C64	1.04211 (6)	0.49309 (12)	0.87715 (13)	0.0304 (3)	
H64	1.044335	0.542326	0.934540	0.036*	
C65	1.07304 (6)	0.40845 (12)	0.88328 (13)	0.0293 (3)	
H65	1.096734	0.398921	0.945407	0.035*	
C57	0.54946 (6)	0.96054 (12)	0.69936 (13)	0.0304 (3)	
H57	0.575278	0.967025	0.643885	0.037*	
C60	0.47446 (7)	0.93988 (13)	0.85612 (13)	0.0329 (3)	
H60	0.448073	0.931561	0.910053	0.040*	
C58	0.53768 (8)	1.04085 (13)	0.76581 (15)	0.0389 (4)	
H58	0.555733	1.102259	0.757872	0.047*	
C59	0.49903 (8)	1.03092 (13)	0.84465 (14)	0.0364 (4)	
H59	0.489588	1.085949	0.890117	0.044*	

### Dipyridinium 4,4'-(1,1,1,3,3,3-hexafluoropropane-2,2-diyl)dibenzoate 4,4'-(1,1,1,3,3,3-hexafluoropropane-2,2-diyl)dibenzoic acid (1) Atomic displacement parameters (Å^2^)

**Table:**

	*U*^11^	*U*^22^	*U*^33^	*U*^12^	*U*^13^	*U*^23^
F12	0.0255 (4)	0.0189 (4)	0.0218 (4)	0.0027 (3)	0.0038 (3)	0.0041 (3)
F17	0.0252 (4)	0.0302 (4)	0.0145 (4)	−0.0009 (3)	0.0046 (3)	−0.0028 (3)
F40	0.0271 (4)	0.0181 (4)	0.0257 (4)	−0.0030 (3)	0.0037 (3)	0.0010 (3)
F44	0.0301 (4)	0.0181 (4)	0.0260 (4)	0.0018 (3)	0.0096 (3)	0.0043 (3)
F13	0.0257 (4)	0.0185 (4)	0.0248 (4)	−0.0017 (3)	0.0006 (3)	−0.0058 (3)
F16	0.0203 (4)	0.0265 (4)	0.0198 (4)	−0.0028 (3)	−0.0032 (3)	−0.0002 (3)
F18	0.0281 (4)	0.0180 (4)	0.0209 (4)	−0.0010 (3)	−0.0014 (3)	0.0033 (3)
F41	0.0296 (4)	0.0221 (4)	0.0217 (4)	0.0034 (3)	0.0029 (3)	−0.0077 (3)
F46	0.0263 (4)	0.0267 (4)	0.0202 (4)	0.0080 (3)	0.0086 (3)	0.0014 (3)
F42	0.0221 (4)	0.0233 (4)	0.0268 (4)	0.0039 (3)	0.0093 (3)	−0.0032 (3)
F45	0.0300 (4)	0.0357 (5)	0.0142 (4)	0.0099 (4)	0.0002 (3)	0.0025 (3)
F39	0.0230 (4)	0.0292 (4)	0.0304 (4)	0.0022 (3)	−0.0078 (3)	−0.0085 (4)
O30	0.0224 (5)	0.0302 (5)	0.0168 (4)	0.0038 (4)	0.0035 (4)	−0.0002 (4)
O3	0.0169 (4)	0.0253 (5)	0.0218 (5)	−0.0046 (4)	0.0000 (4)	−0.0002 (4)
O1	0.0236 (5)	0.0272 (5)	0.0210 (5)	−0.0019 (4)	−0.0041 (4)	0.0005 (4)
O27	0.0184 (5)	0.0335 (5)	0.0284 (5)	0.0060 (4)	0.0092 (4)	0.0061 (4)
O53	0.0246 (5)	0.0287 (5)	0.0259 (5)	0.0059 (4)	−0.0046 (4)	−0.0005 (4)
O28	0.0198 (5)	0.0287 (5)	0.0274 (5)	0.0070 (4)	0.0082 (4)	0.0064 (4)
O55	0.0189 (5)	0.0302 (5)	0.0323 (6)	−0.0024 (4)	−0.0031 (4)	−0.0054 (4)
N56	0.0218 (6)	0.0274 (6)	0.0181 (5)	0.0059 (5)	0.0011 (4)	0.0010 (5)
N62	0.0187 (5)	0.0299 (6)	0.0209 (6)	−0.0022 (5)	0.0000 (4)	0.0053 (5)
O26	0.0351 (7)	0.0400 (7)	0.1084 (13)	−0.0065 (6)	0.0369 (8)	−0.0330 (8)
C47	0.0155 (6)	0.0186 (6)	0.0148 (6)	0.0005 (5)	0.0030 (5)	0.0019 (5)
C31	0.0133 (6)	0.0196 (6)	0.0161 (6)	−0.0017 (5)	0.0002 (5)	−0.0034 (5)
C7	0.0160 (6)	0.0161 (6)	0.0169 (6)	0.0015 (5)	0.0018 (5)	−0.0006 (5)
C52	0.0186 (6)	0.0166 (6)	0.0207 (6)	0.0020 (5)	0.0031 (5)	−0.0001 (5)
C32	0.0157 (6)	0.0171 (6)	0.0179 (6)	0.0002 (5)	−0.0010 (5)	0.0006 (5)
C19	0.0151 (6)	0.0194 (6)	0.0142 (6)	0.0014 (5)	0.0007 (5)	−0.0001 (5)
C29	0.0140 (6)	0.0209 (6)	0.0190 (6)	−0.0018 (5)	0.0005 (5)	−0.0017 (5)
C36	0.0179 (6)	0.0221 (6)	0.0146 (6)	−0.0009 (5)	0.0010 (5)	0.0003 (5)
C33	0.0178 (6)	0.0181 (6)	0.0147 (6)	−0.0003 (5)	0.0005 (5)	0.0007 (5)
C4	0.0161 (6)	0.0171 (6)	0.0189 (6)	0.0018 (5)	0.0019 (5)	−0.0014 (5)
C35	0.0193 (6)	0.0178 (6)	0.0164 (6)	0.0023 (5)	−0.0005 (5)	0.0006 (5)
C34	0.0153 (6)	0.0173 (6)	0.0145 (6)	−0.0001 (5)	0.0011 (5)	−0.0023 (5)
C50	0.0148 (6)	0.0219 (6)	0.0177 (6)	−0.0010 (5)	0.0036 (5)	0.0021 (5)
C48	0.0195 (6)	0.0178 (6)	0.0181 (6)	0.0006 (5)	0.0032 (5)	−0.0014 (5)
C49	0.0174 (6)	0.0188 (6)	0.0199 (6)	0.0029 (5)	0.0034 (5)	0.0015 (5)
C8	0.0195 (6)	0.0229 (6)	0.0142 (6)	−0.0009 (5)	0.0024 (5)	0.0000 (5)
C51	0.0188 (6)	0.0194 (6)	0.0194 (6)	−0.0022 (5)	0.0027 (5)	−0.0028 (5)
C2	0.0173 (6)	0.0177 (6)	0.0208 (6)	0.0019 (5)	0.0018 (5)	−0.0025 (5)
C24	0.0186 (6)	0.0174 (6)	0.0197 (6)	−0.0007 (5)	0.0016 (5)	0.0001 (5)
C37	0.0186 (6)	0.0158 (6)	0.0145 (6)	0.0023 (5)	0.0011 (5)	−0.0002 (5)
C11	0.0182 (6)	0.0188 (6)	0.0188 (6)	0.0003 (5)	0.0032 (5)	−0.0005 (5)
C9	0.0186 (6)	0.0212 (6)	0.0191 (6)	−0.0016 (5)	0.0043 (5)	0.0003 (5)
C5	0.0198 (6)	0.0225 (6)	0.0153 (6)	0.0010 (5)	0.0009 (5)	0.0007 (5)
C22	0.0156 (6)	0.0275 (7)	0.0169 (6)	0.0021 (5)	0.0000 (5)	−0.0023 (5)
C20	0.0173 (6)	0.0201 (6)	0.0213 (6)	−0.0016 (5)	0.0010 (5)	−0.0024 (5)
C6	0.0179 (6)	0.0211 (6)	0.0167 (6)	−0.0004 (5)	0.0035 (5)	0.0014 (5)
C38	0.0207 (6)	0.0198 (6)	0.0182 (6)	0.0024 (5)	−0.0001 (5)	−0.0024 (5)
C54	0.0146 (6)	0.0271 (7)	0.0194 (6)	−0.0009 (5)	0.0031 (5)	−0.0005 (5)
C15	0.0185 (6)	0.0192 (6)	0.0170 (6)	−0.0013 (5)	0.0015 (5)	−0.0020 (5)
C10	0.0174 (6)	0.0165 (6)	0.0150 (6)	−0.0007 (5)	0.0030 (5)	−0.0005 (5)
C23	0.0164 (6)	0.0236 (7)	0.0188 (6)	−0.0009 (5)	0.0008 (5)	0.0019 (5)
C67	0.0219 (7)	0.0254 (7)	0.0220 (7)	−0.0049 (5)	0.0026 (5)	0.0012 (6)
C21	0.0191 (6)	0.0220 (7)	0.0244 (7)	0.0021 (5)	0.0012 (5)	−0.0062 (5)
C43	0.0222 (6)	0.0202 (6)	0.0173 (6)	0.0054 (5)	0.0040 (5)	0.0001 (5)
C66	0.0215 (7)	0.0289 (7)	0.0268 (7)	−0.0002 (6)	0.0006 (6)	0.0067 (6)
C25	0.0179 (6)	0.0338 (8)	0.0254 (7)	0.0005 (6)	0.0035 (5)	−0.0073 (6)
C63	0.0273 (7)	0.0259 (7)	0.0291 (8)	0.0005 (6)	0.0076 (6)	0.0034 (6)
C61	0.0260 (7)	0.0338 (8)	0.0244 (7)	−0.0001 (6)	0.0040 (6)	0.0019 (6)
C64	0.0341 (8)	0.0328 (8)	0.0246 (7)	−0.0089 (6)	0.0041 (6)	−0.0037 (6)
C65	0.0244 (7)	0.0414 (9)	0.0218 (7)	−0.0064 (6)	−0.0029 (6)	0.0057 (6)
C57	0.0332 (8)	0.0311 (8)	0.0273 (8)	0.0009 (6)	0.0065 (6)	0.0064 (6)
C60	0.0348 (8)	0.0408 (9)	0.0237 (8)	0.0076 (7)	0.0089 (6)	0.0014 (7)
C58	0.0568 (11)	0.0266 (8)	0.0334 (9)	−0.0012 (7)	0.0069 (8)	0.0043 (7)
C59	0.0542 (11)	0.0309 (8)	0.0242 (8)	0.0140 (7)	0.0034 (7)	0.0005 (6)

### Dipyridinium 4,4'-(1,1,1,3,3,3-hexafluoropropane-2,2-diyl)dibenzoate 4,4'-(1,1,1,3,3,3-hexafluoropropane-2,2-diyl)dibenzoic acid (1) Geometric parameters (Å, º)

**Table:**

F12—C11	1.3392 (16)	C4—C9	1.3934 (19)
F17—C15	1.3429 (15)	C4—C5	1.3923 (19)
F40—C38	1.3365 (16)	C35—H35	0.9500
F44—C43	1.3357 (16)	C35—C34	1.4005 (18)
F13—C11	1.3419 (15)	C34—C37	1.5446 (17)
F16—C15	1.3392 (15)	C50—C49	1.3886 (19)
F18—C15	1.3359 (15)	C50—C51	1.3922 (19)
F41—C38	1.3404 (16)	C50—C54	1.5085 (18)
F46—C43	1.3441 (15)	C48—H48	0.9500
F42—C11	1.3367 (15)	C48—C49	1.3909 (19)
F45—C43	1.3407 (16)	C49—H49	0.9500
F39—C38	1.3399 (15)	C8—H8	0.9500
O30—C29	1.2602 (17)	C8—C9	1.3873 (19)
O3—H3	0.8400	C51—H51	0.9500
O3—C2	1.3209 (17)	C24—H24	0.9500
O1—C2	1.2152 (17)	C24—C23	1.3889 (19)
O27—H27	0.8400	C37—C38	1.5502 (18)
O27—C25	1.3073 (18)	C37—C43	1.5460 (18)
O53—C54	1.2680 (18)	C11—C10	1.5463 (18)
O28—C29	1.2596 (17)	C9—H9	0.9500
O55—C54	1.2507 (18)	C5—H5	0.9500
N56—H56	0.8800	C5—C6	1.3880 (19)
N56—C61	1.3371 (19)	C22—C23	1.390 (2)
N56—C57	1.330 (2)	C22—C21	1.391 (2)
N62—H62	0.8800	C22—C25	1.4968 (19)
N62—C67	1.3383 (19)	C20—H20	0.9500
N62—C63	1.339 (2)	C20—C21	1.3875 (19)
O26—C25	1.217 (2)	C6—H6	0.9500
C47—C52	1.3991 (18)	C15—C10	1.5532 (18)
C47—C48	1.3941 (18)	C23—H23	0.9500
C47—C37	1.5448 (17)	C67—H67	0.9500
C31—C32	1.3920 (18)	C67—C66	1.381 (2)
C31—C29	1.5113 (18)	C21—H21	0.9500
C31—C36	1.3935 (19)	C66—H66	0.9500
C7—C8	1.3965 (18)	C66—C65	1.384 (2)
C7—C6	1.3973 (18)	C63—H63	0.9500
C7—C10	1.5387 (17)	C63—C64	1.378 (2)
C52—H52	0.9500	C61—H61	0.9500
C52—C51	1.3865 (19)	C61—C60	1.375 (2)
C32—H32	0.9500	C64—H64	0.9500
C32—C33	1.3889 (18)	C64—C65	1.384 (2)
C19—C24	1.3941 (18)	C65—H65	0.9500
C19—C20	1.3931 (19)	C57—H57	0.9500
C19—C10	1.5443 (17)	C57—C58	1.374 (2)
C36—H36	0.9500	C60—H60	0.9500
C36—C35	1.3875 (18)	C60—C59	1.381 (3)
C33—H33	0.9500	C58—H58	0.9500
C33—C34	1.3936 (18)	C58—C59	1.387 (3)
C4—C2	1.4991 (18)	C59—H59	0.9500
			
C2—O3—H3	109.5	C8—C9—H9	119.8
C25—O27—H27	109.5	C4—C5—H5	119.8
C61—N56—H56	119.2	C6—C5—C4	120.31 (12)
C57—N56—H56	119.2	C6—C5—H5	119.8
C57—N56—C61	121.67 (13)	C23—C22—C21	119.76 (12)
C67—N62—H62	119.6	C23—C22—C25	121.87 (13)
C67—N62—C63	120.81 (13)	C21—C22—C25	118.35 (13)
C63—N62—H62	119.6	C19—C20—H20	119.9
C52—C47—C37	117.58 (11)	C21—C20—C19	120.27 (12)
C48—C47—C52	118.96 (12)	C21—C20—H20	119.9
C48—C47—C37	123.44 (11)	C7—C6—H6	119.7
C32—C31—C29	120.22 (12)	C5—C6—C7	120.65 (12)
C32—C31—C36	118.80 (12)	C5—C6—H6	119.7
C36—C31—C29	120.99 (12)	F40—C38—F41	106.99 (10)
C8—C7—C6	118.78 (12)	F40—C38—F39	106.84 (11)
C8—C7—C10	123.03 (11)	F40—C38—C37	111.26 (10)
C6—C7—C10	118.09 (11)	F41—C38—C37	113.57 (11)
C47—C52—H52	119.7	F39—C38—F41	106.39 (10)
C51—C52—C47	120.54 (12)	F39—C38—C37	111.39 (10)
C51—C52—H52	119.7	O53—C54—C50	116.42 (12)
C31—C32—H32	119.5	O55—C54—O53	125.79 (12)
C33—C32—C31	120.97 (12)	O55—C54—C50	117.79 (12)
C33—C32—H32	119.5	F17—C15—C10	113.00 (11)
C24—C19—C10	123.12 (11)	F16—C15—F17	106.31 (10)
C20—C19—C24	119.26 (12)	F16—C15—C10	111.83 (11)
C20—C19—C10	117.34 (11)	F18—C15—F17	107.19 (11)
O30—C29—C31	117.70 (12)	F18—C15—F16	107.03 (10)
O28—C29—O30	125.45 (12)	F18—C15—C10	111.14 (10)
O28—C29—C31	116.85 (12)	C7—C10—C19	112.24 (10)
C31—C36—H36	119.8	C7—C10—C11	106.77 (10)
C35—C36—C31	120.47 (12)	C7—C10—C15	112.39 (10)
C35—C36—H36	119.8	C19—C10—C11	112.36 (10)
C32—C33—H33	119.8	C19—C10—C15	105.00 (10)
C32—C33—C34	120.35 (12)	C11—C10—C15	108.10 (10)
C34—C33—H33	119.8	C24—C23—C22	119.97 (13)
C9—C4—C2	121.32 (12)	C24—C23—H23	120.0
C5—C4—C2	119.43 (12)	C22—C23—H23	120.0
C5—C4—C9	119.25 (12)	N62—C67—H67	119.6
C36—C35—H35	119.6	N62—C67—C66	120.89 (14)
C36—C35—C34	120.71 (12)	C66—C67—H67	119.6
C34—C35—H35	119.6	C22—C21—H21	119.9
C33—C34—C35	118.69 (12)	C20—C21—C22	120.23 (13)
C33—C34—C37	123.97 (11)	C20—C21—H21	119.9
C35—C34—C37	117.29 (11)	F44—C43—F46	106.43 (11)
C49—C50—C51	119.40 (12)	F44—C43—F45	107.53 (11)
C49—C50—C54	120.82 (12)	F44—C43—C37	111.05 (11)
C51—C50—C54	119.77 (12)	F46—C43—C37	111.60 (11)
C47—C48—H48	119.9	F45—C43—F46	106.54 (10)
C49—C48—C47	120.22 (12)	F45—C43—C37	113.33 (11)
C49—C48—H48	119.9	C67—C66—H66	120.6
C50—C49—C48	120.55 (12)	C67—C66—C65	118.71 (14)
C50—C49—H49	119.7	C65—C66—H66	120.6
C48—C49—H49	119.7	O27—C25—C22	113.69 (13)
C7—C8—H8	119.8	O26—C25—O27	124.54 (14)
C9—C8—C7	120.49 (12)	O26—C25—C22	121.72 (14)
C9—C8—H8	119.8	N62—C63—H63	119.5
C52—C51—C50	120.23 (12)	N62—C63—C64	121.10 (14)
C52—C51—H51	119.9	C64—C63—H63	119.5
C50—C51—H51	119.9	N56—C61—H61	120.0
O3—C2—C4	112.03 (11)	N56—C61—C60	120.06 (15)
O1—C2—O3	125.01 (12)	C60—C61—H61	120.0
O1—C2—C4	122.97 (12)	C63—C64—H64	120.7
C19—C24—H24	119.8	C63—C64—C65	118.65 (14)
C23—C24—C19	120.48 (12)	C65—C64—H64	120.7
C23—C24—H24	119.8	C66—C65—H65	120.1
C47—C37—C38	112.44 (10)	C64—C65—C66	119.83 (14)
C47—C37—C43	106.48 (10)	C64—C65—H65	120.1
C34—C37—C47	110.64 (10)	N56—C57—H57	119.7
C34—C37—C38	106.14 (10)	N56—C57—C58	120.64 (15)
C34—C37—C43	113.24 (10)	C58—C57—H57	119.7
C43—C37—C38	107.98 (10)	C61—C60—H60	120.2
F12—C11—F13	107.41 (10)	C61—C60—C59	119.52 (15)
F12—C11—C10	111.19 (11)	C59—C60—H60	120.2
F13—C11—C10	112.53 (11)	C57—C58—H58	120.5
F42—C11—F12	106.59 (10)	C57—C58—C59	119.01 (16)
F42—C11—F13	106.93 (10)	C59—C58—H58	120.5
F42—C11—C10	111.87 (10)	C60—C59—C58	119.07 (15)
C4—C9—H9	119.8	C60—C59—H59	120.5
C8—C9—C4	120.48 (12)	C58—C59—H59	120.5
			
F12—C11—C10—C7	−73.81 (13)	C48—C47—C52—C51	−2.13 (19)
F12—C11—C10—C19	49.66 (14)	C48—C47—C37—C34	142.26 (12)
F12—C11—C10—C15	165.06 (10)	C48—C47—C37—C38	23.77 (17)
F17—C15—C10—C7	−71.96 (13)	C48—C47—C37—C43	−94.29 (14)
F17—C15—C10—C19	165.75 (10)	C49—C50—C51—C52	2.7 (2)
F17—C15—C10—C11	45.62 (14)	C49—C50—C54—O53	−13.18 (19)
F13—C11—C10—C7	165.65 (10)	C49—C50—C54—O55	167.73 (13)
F13—C11—C10—C19	−70.88 (14)	C8—C7—C6—C5	−0.79 (19)
F13—C11—C10—C15	44.51 (14)	C8—C7—C10—C19	141.09 (12)
F16—C15—C10—C7	168.13 (10)	C8—C7—C10—C11	−95.37 (14)
F16—C15—C10—C19	45.84 (13)	C8—C7—C10—C15	23.00 (17)
F16—C15—C10—C11	−74.29 (13)	C51—C50—C49—C48	−1.5 (2)
F18—C15—C10—C7	48.59 (14)	C51—C50—C54—O53	165.83 (12)
F18—C15—C10—C19	−73.71 (13)	C51—C50—C54—O55	−13.26 (19)
F18—C15—C10—C11	166.17 (10)	C2—C4—C9—C8	−179.35 (12)
F42—C11—C10—C7	45.24 (14)	C2—C4—C5—C6	−179.14 (12)
F42—C11—C10—C19	168.71 (10)	C24—C19—C20—C21	1.51 (19)
F42—C11—C10—C15	−75.90 (13)	C24—C19—C10—C7	141.61 (12)
N56—C61—C60—C59	−1.0 (2)	C24—C19—C10—C11	21.26 (17)
N56—C57—C58—C59	−1.5 (3)	C24—C19—C10—C15	−96.00 (14)
N62—C67—C66—C65	0.4 (2)	C37—C47—C52—C51	179.52 (12)
N62—C63—C64—C65	0.0 (2)	C37—C47—C48—C49	−178.47 (12)
C47—C52—C51—C50	−0.9 (2)	C9—C4—C2—O3	−15.59 (17)
C47—C48—C49—C50	−1.5 (2)	C9—C4—C2—O1	163.94 (13)
C47—C37—C38—F40	50.14 (14)	C9—C4—C5—C6	1.4 (2)
C47—C37—C38—F41	−70.65 (14)	C5—C4—C2—O3	164.93 (12)
C47—C37—C38—F39	169.24 (11)	C5—C4—C2—O1	−15.6 (2)
C47—C37—C43—F44	−67.10 (13)	C5—C4—C9—C8	0.1 (2)
C47—C37—C43—F46	51.47 (14)	C20—C19—C24—C23	−0.35 (19)
C47—C37—C43—F45	171.75 (10)	C20—C19—C10—C7	−44.43 (15)
C31—C32—C33—C34	−1.22 (19)	C20—C19—C10—C11	−164.79 (11)
C31—C36—C35—C34	−0.94 (19)	C20—C19—C10—C15	77.96 (13)
C7—C8—C9—C4	−2.0 (2)	C6—C7—C8—C9	2.29 (19)
C52—C47—C48—C49	3.29 (19)	C6—C7—C10—C19	−42.64 (15)
C52—C47—C37—C34	−39.47 (15)	C6—C7—C10—C11	80.90 (14)
C52—C47—C37—C38	−157.96 (11)	C6—C7—C10—C15	−160.73 (11)
C52—C47—C37—C43	83.97 (14)	C38—C37—C43—F44	171.93 (10)
C32—C31—C29—O30	−176.34 (12)	C38—C37—C43—F46	−69.49 (13)
C32—C31—C29—O28	3.42 (18)	C38—C37—C43—F45	50.78 (14)
C32—C31—C36—C35	0.44 (19)	C54—C50—C49—C48	177.47 (12)
C32—C33—C34—C35	0.71 (19)	C54—C50—C51—C52	−176.32 (12)
C32—C33—C34—C37	178.22 (12)	C10—C7—C8—C9	178.54 (12)
C19—C24—C23—C22	−0.9 (2)	C10—C7—C6—C5	−177.23 (12)
C19—C20—C21—C22	−1.4 (2)	C10—C19—C24—C23	173.49 (12)
C29—C31—C32—C33	−178.96 (11)	C10—C19—C20—C21	−172.68 (12)
C29—C31—C36—C35	−179.97 (12)	C23—C22—C21—C20	0.1 (2)
C36—C31—C32—C33	0.64 (19)	C23—C22—C25—O27	−7.03 (19)
C36—C31—C29—O30	4.08 (18)	C23—C22—C25—O26	170.47 (16)
C36—C31—C29—O28	−176.17 (12)	C67—N62—C63—C64	0.0 (2)
C36—C35—C34—C33	0.36 (19)	C67—C66—C65—C64	−0.4 (2)
C36—C35—C34—C37	−177.31 (11)	C21—C22—C23—C24	1.1 (2)
C33—C34—C37—C47	133.38 (13)	C21—C22—C25—O27	174.16 (12)
C33—C34—C37—C38	−104.36 (14)	C21—C22—C25—O26	−8.3 (2)
C33—C34—C37—C43	13.94 (17)	C43—C37—C38—F40	167.32 (10)
C4—C5—C6—C7	−1.0 (2)	C43—C37—C38—F41	46.52 (14)
C35—C34—C37—C47	−49.08 (15)	C43—C37—C38—F39	−73.59 (13)
C35—C34—C37—C38	73.18 (14)	C25—C22—C23—C24	−177.72 (13)
C35—C34—C37—C43	−168.52 (11)	C25—C22—C21—C20	178.93 (13)
C34—C37—C38—F40	−70.96 (13)	C63—N62—C67—C66	−0.2 (2)
C34—C37—C38—F41	168.25 (10)	C63—C64—C65—C66	0.2 (2)
C34—C37—C38—F39	48.14 (14)	C61—N56—C57—C58	0.0 (2)
C34—C37—C43—F44	54.71 (14)	C61—C60—C59—C58	−0.5 (3)
C34—C37—C43—F46	173.28 (10)	C57—N56—C61—C60	1.3 (2)
C34—C37—C43—F45	−66.44 (14)	C57—C58—C59—C60	1.7 (3)

### Dipyridinium 4,4'-(1,1,1,3,3,3-hexafluoropropane-2,2-diyl)dibenzoate 4,4'-(1,1,1,3,3,3-hexafluoropropane-2,2-diyl)dibenzoic acid (1) Hydrogen-bond geometry (Å, º)

**Table:**

*D*—H···*A*	*D*—H	H···*A*	*D*···*A*	*D*—H···*A*
O3—H3···O55^i^	0.84	1.75	2.5732 (13)	164
O27—H27···O28	0.84	1.68	2.5125 (13)	172
N56—H56···O30	0.88	1.79	2.6488 (15)	165
N62—H62···O53	0.88	1.67	2.5472 (15)	177

Symmetry code: (i) *x*−1, *y*, *z*.

### Ethane-1,2-diaminium 4,4'-(1,1,1,3,3,3-hexafluoropropane-2,2-diyl)dibenzoate monohydrate (2) Crystal data

**Table:**

C_2_H_10_N_2_^2+^·C_17_H_8_F_6_O_4_^2^^−^·H_2_O	*D*_x_ = 1.498 Mg m^−^^3^
*M**_r_* = 470.37	Cu *K*α radiation, λ = 1.54184 Å
Orthorhombic, *P**b**c**a*	Cell parameters from 9861 reflections
*a* = 13.2518 (3) Å	θ = 3.4–75.7°
*b* = 12.1773 (3) Å	µ = 1.26 mm^−^^1^
*c* = 25.8419 (6) Å	*T* = 93 K
*V* = 4170.14 (17) Å^3^	Needle, colourless
*Z* = 8	0.22 × 0.1 × 0.05 mm
*F*(000) = 1936	

### Ethane-1,2-diaminium 4,4'-(1,1,1,3,3,3-hexafluoropropane-2,2-diyl)dibenzoate monohydrate (2) Data collection

**Table:**

XtaLAB Synergy R, DW system, HyPix diffractometer	4198 independent reflections
Radiation source: Rotating-anode X-ray tube, Rigaku (Cu) X-ray Source	3809 reflections with *I* > 2σ(*I*)
Mirror monochromator	*R*_int_ = 0.029
Detector resolution: 10.0000 pixels mm^-1^	θ_max_ = 76.3°, θ_min_ = 3.4°
ω scans	*h* = −6→16
Absorption correction: multi-scan (CrysAlisPro; Rigaku OD, 2019)	*k* = −14→15
*T*_min_ = 0.855, *T*_max_ = 0.940	*l* = −32→29
15896 measured reflections	

### Ethane-1,2-diaminium 4,4'-(1,1,1,3,3,3-hexafluoropropane-2,2-diyl)dibenzoate monohydrate (2) Refinement

**Table:**

Refinement on *F*^2^	Primary atom site location: dual
Least-squares matrix: full	Hydrogen site location: mixed
*R*[*F*^2^ > 2σ(*F*^2^)] = 0.032	H-atom parameters constrained
*wR*(*F*^2^) = 0.082	*w* = 1/[σ^2^(*F*_o_^2^) + (0.0378*P*)^2^ + 1.7828*P*] where *P* = (*F*_o_^2^ + 2*F*_c_^2^)/3
*S* = 1.04	(Δ/σ)_max_ < 0.001
4198 reflections	Δρ_max_ = 0.29 e Å^−^^3^
294 parameters	Δρ_min_ = −0.24 e Å^−^^3^
0 restraints	

### Ethane-1,2-diaminium 4,4'-(1,1,1,3,3,3-hexafluoropropane-2,2-diyl)dibenzoate monohydrate (2) Special details

**Table:**

Geometry. All esds (except the esd in the dihedral angle between two l.s. planes) are estimated using the full covariance matrix. The cell esds are taken into account individually in the estimation of esds in distances, angles and torsion angles; correlations between esds in cell parameters are only used when they are defined by crystal symmetry. An approximate (isotropic) treatment of cell esds is used for estimating esds involving l.s. planes.

### Ethane-1,2-diaminium 4,4'-(1,1,1,3,3,3-hexafluoropropane-2,2-diyl)dibenzoate monohydrate (2) Fractional atomic coordinates and isotropic or equivalent isotropic displacement parameters (Å^2^)

**Table:**

	*x*	*y*	*z*	*U*_iso_*/*U*_eq_	
F18	0.40013 (5)	0.38652 (6)	0.26137 (3)	0.02225 (17)	
F16	0.40185 (6)	0.53316 (6)	0.21406 (3)	0.02269 (17)	
F14	0.71228 (6)	0.55005 (6)	0.21542 (3)	0.02310 (17)	
F13	0.59106 (6)	0.64981 (6)	0.24516 (3)	0.02445 (18)	
F12	0.58145 (6)	0.58279 (6)	0.16850 (3)	0.02503 (18)	
F17	0.44371 (6)	0.54163 (7)	0.29429 (3)	0.02490 (18)	
O26	0.81858 (7)	0.35414 (7)	0.44805 (3)	0.01964 (19)	
O27	0.72698 (7)	0.20072 (7)	0.44559 (4)	0.0221 (2)	
O3	0.70242 (7)	0.09542 (7)	0.06295 (4)	0.0212 (2)	
O1	0.54140 (7)	0.11490 (8)	0.04076 (4)	0.0276 (2)	
O32	1.07320 (7)	0.45205 (8)	0.56164 (5)	0.0328 (3)	
H32A	1.119538	0.404618	0.557451	0.049*	
H32B	1.104051	0.513230	0.562216	0.049*	
N28	0.85940 (8)	0.09566 (8)	0.51156 (4)	0.0179 (2)	
H28A	0.918390	0.094540	0.493539	0.022*	
H28B	0.813704	0.138226	0.494574	0.022*	
H28C	0.834993	0.026082	0.514447	0.022*	
N31	0.89649 (8)	0.34315 (9)	0.54417 (4)	0.0184 (2)	
H31A	0.880147	0.333232	0.510285	0.022*	
H31B	0.940104	0.400534	0.547105	0.022*	
H31C	0.839576	0.357676	0.562642	0.022*	
C25	0.75141 (9)	0.29400 (10)	0.42854 (5)	0.0159 (2)	
C24	0.60904 (9)	0.30292 (10)	0.30102 (5)	0.0162 (2)	
H24	0.576891	0.252964	0.278094	0.019*	
C8	0.66525 (9)	0.31708 (10)	0.18675 (5)	0.0164 (2)	
H8	0.719685	0.333736	0.209351	0.020*	
C23	0.65162 (9)	0.26430 (10)	0.34664 (5)	0.0165 (2)	
H23	0.648009	0.188315	0.354774	0.020*	
C19	0.61300 (9)	0.41468 (10)	0.28846 (5)	0.0147 (2)	
C7	0.57178 (9)	0.36773 (10)	0.19423 (5)	0.0151 (2)	
C9	0.67956 (9)	0.24269 (10)	0.14669 (5)	0.0166 (2)	
H9	0.743403	0.208360	0.142465	0.020*	
C22	0.69956 (9)	0.33629 (10)	0.38054 (5)	0.0157 (2)	
C10	0.56220 (9)	0.45362 (10)	0.23788 (5)	0.0154 (2)	
C4	0.60139 (9)	0.21785 (10)	0.11266 (5)	0.0170 (2)	
C15	0.45109 (9)	0.47935 (10)	0.25180 (5)	0.0181 (2)	
C21	0.70136 (10)	0.44798 (10)	0.36872 (5)	0.0186 (3)	
H21	0.732850	0.497929	0.391889	0.022*	
C2	0.61579 (9)	0.13734 (10)	0.06870 (5)	0.0186 (3)	
C20	0.65761 (10)	0.48710 (10)	0.32345 (5)	0.0192 (3)	
H20	0.658091	0.563621	0.316301	0.023*	
C11	0.61193 (10)	0.56012 (10)	0.21695 (5)	0.0188 (3)	
C6	0.49324 (9)	0.34242 (11)	0.16015 (5)	0.0199 (3)	
H6	0.429192	0.376328	0.164335	0.024*	
C5	0.50841 (10)	0.26791 (11)	0.12021 (5)	0.0213 (3)	
H5	0.454093	0.250860	0.097600	0.026*	
C29	0.87763 (10)	0.14176 (11)	0.56419 (5)	0.0219 (3)	
H29A	0.908692	0.084227	0.586073	0.026*	
H29B	0.811902	0.161441	0.579868	0.026*	
C30	0.94494 (10)	0.24182 (11)	0.56454 (5)	0.0215 (3)	
H30A	0.967314	0.255690	0.600507	0.026*	
H30B	1.005784	0.225943	0.543609	0.026*	

### Ethane-1,2-diaminium 4,4'-(1,1,1,3,3,3-hexafluoropropane-2,2-diyl)dibenzoate monohydrate (2) Atomic displacement parameters (Å^2^)

**Table:**

	*U*^11^	*U*^22^	*U*^33^	*U*^12^	*U*^13^	*U*^23^
F18	0.0171 (4)	0.0199 (4)	0.0298 (4)	−0.0018 (3)	0.0034 (3)	0.0003 (3)
F16	0.0201 (4)	0.0194 (4)	0.0285 (4)	0.0061 (3)	−0.0062 (3)	−0.0004 (3)
F14	0.0188 (4)	0.0218 (4)	0.0287 (4)	−0.0052 (3)	−0.0009 (3)	0.0058 (3)
F13	0.0315 (4)	0.0111 (3)	0.0307 (4)	0.0002 (3)	−0.0068 (3)	0.0003 (3)
F12	0.0320 (4)	0.0213 (4)	0.0217 (4)	−0.0043 (3)	−0.0072 (3)	0.0082 (3)
F17	0.0224 (4)	0.0258 (4)	0.0264 (4)	0.0056 (3)	−0.0003 (3)	−0.0101 (3)
O26	0.0198 (4)	0.0202 (4)	0.0190 (4)	−0.0032 (3)	−0.0036 (3)	0.0016 (4)
O27	0.0245 (5)	0.0179 (4)	0.0239 (5)	−0.0030 (3)	−0.0061 (4)	0.0062 (4)
O3	0.0185 (4)	0.0196 (4)	0.0256 (5)	−0.0009 (3)	0.0023 (4)	−0.0055 (4)
O1	0.0262 (5)	0.0276 (5)	0.0291 (5)	0.0041 (4)	−0.0101 (4)	−0.0104 (4)
O32	0.0206 (5)	0.0177 (5)	0.0600 (7)	−0.0011 (4)	−0.0056 (5)	−0.0012 (5)
N28	0.0184 (5)	0.0154 (5)	0.0199 (5)	0.0003 (4)	−0.0013 (4)	0.0012 (4)
N31	0.0189 (5)	0.0173 (5)	0.0190 (5)	0.0002 (4)	−0.0022 (4)	−0.0007 (4)
C25	0.0147 (5)	0.0164 (5)	0.0165 (5)	0.0016 (4)	0.0011 (5)	−0.0007 (5)
C24	0.0171 (6)	0.0143 (6)	0.0171 (6)	−0.0022 (4)	−0.0002 (4)	−0.0008 (5)
C8	0.0156 (6)	0.0174 (6)	0.0164 (6)	−0.0011 (4)	−0.0016 (4)	0.0014 (5)
C23	0.0185 (6)	0.0125 (5)	0.0186 (6)	−0.0008 (4)	0.0013 (5)	0.0008 (4)
C19	0.0144 (5)	0.0143 (5)	0.0152 (6)	0.0006 (4)	0.0008 (4)	0.0008 (4)
C7	0.0180 (6)	0.0126 (5)	0.0146 (5)	−0.0006 (4)	0.0001 (4)	0.0018 (4)
C9	0.0152 (5)	0.0169 (6)	0.0177 (6)	0.0010 (4)	0.0005 (4)	0.0023 (5)
C22	0.0133 (5)	0.0175 (6)	0.0163 (6)	0.0007 (4)	0.0015 (4)	0.0014 (5)
C10	0.0151 (5)	0.0123 (5)	0.0186 (6)	0.0007 (4)	−0.0013 (4)	0.0009 (4)
C4	0.0198 (6)	0.0139 (5)	0.0173 (6)	−0.0002 (4)	−0.0010 (5)	0.0011 (5)
C15	0.0179 (6)	0.0144 (5)	0.0219 (6)	0.0014 (5)	−0.0018 (5)	−0.0021 (5)
C21	0.0210 (6)	0.0156 (6)	0.0192 (6)	−0.0015 (5)	−0.0032 (5)	−0.0018 (5)
C2	0.0205 (6)	0.0155 (6)	0.0199 (6)	−0.0009 (5)	−0.0005 (5)	0.0003 (5)
C20	0.0233 (6)	0.0130 (6)	0.0214 (6)	−0.0001 (5)	−0.0028 (5)	0.0007 (5)
C11	0.0211 (6)	0.0156 (6)	0.0197 (6)	−0.0012 (5)	−0.0036 (5)	0.0030 (5)
C6	0.0165 (6)	0.0205 (6)	0.0228 (6)	0.0043 (5)	−0.0036 (5)	−0.0027 (5)
C5	0.0201 (6)	0.0215 (6)	0.0223 (6)	0.0018 (5)	−0.0074 (5)	−0.0035 (5)
C29	0.0291 (7)	0.0186 (6)	0.0181 (6)	0.0013 (5)	0.0005 (5)	0.0006 (5)
C30	0.0215 (6)	0.0198 (6)	0.0233 (6)	0.0036 (5)	−0.0064 (5)	−0.0012 (5)

### Ethane-1,2-diaminium 4,4'-(1,1,1,3,3,3-hexafluoropropane-2,2-diyl)dibenzoate monohydrate (2) Geometric parameters (Å, º)

**Table:**

F18—C15	1.3398 (14)	C8—C9	1.3886 (18)
F16—C15	1.3439 (15)	C23—H23	0.9500
F14—C11	1.3361 (15)	C23—C22	1.3926 (17)
F13—C11	1.3419 (15)	C19—C10	1.5447 (16)
F12—C11	1.3442 (15)	C19—C20	1.3946 (18)
F17—C15	1.3381 (15)	C7—C10	1.5436 (17)
O26—C25	1.2580 (15)	C7—C6	1.3977 (17)
O27—C25	1.2607 (15)	C9—H9	0.9500
O3—C2	1.2652 (16)	C9—C4	1.3922 (17)
O1—C2	1.2520 (16)	C22—C21	1.3941 (17)
O32—H32A	0.8499	C10—C15	1.5478 (17)
O32—H32B	0.8500	C10—C11	1.5520 (17)
N28—H28A	0.9100	C4—C2	1.5127 (17)
N28—H28B	0.9100	C4—C5	1.3884 (18)
N28—H28C	0.9100	C21—H21	0.9500
N28—C29	1.4911 (17)	C21—C20	1.3900 (18)
N31—H31A	0.9100	C20—H20	0.9500
N31—H31B	0.9100	C6—H6	0.9500
N31—H31C	0.9100	C6—C5	1.3890 (18)
N31—C30	1.4873 (16)	C5—H5	0.9500
C25—C22	1.5086 (16)	C29—H29A	0.9900
C24—H24	0.9500	C29—H29B	0.9900
C24—C23	1.3891 (18)	C29—C30	1.5102 (18)
C24—C19	1.4001 (17)	C30—H30A	0.9900
C8—H8	0.9500	C30—H30B	0.9900
C8—C7	1.3972 (17)		
			
H32A—O32—H32B	104.5	C9—C4—C2	121.43 (11)
H28A—N28—H28B	109.5	C5—C4—C9	118.44 (11)
H28A—N28—H28C	109.5	C5—C4—C2	120.13 (11)
H28B—N28—H28C	109.5	F18—C15—F16	107.50 (10)
C29—N28—H28A	109.5	F18—C15—C10	110.58 (9)
C29—N28—H28B	109.5	F16—C15—C10	113.08 (10)
C29—N28—H28C	109.5	F17—C15—F18	106.85 (10)
H31A—N31—H31B	109.5	F17—C15—F16	106.48 (9)
H31A—N31—H31C	109.5	F17—C15—C10	112.02 (10)
H31B—N31—H31C	109.5	C22—C21—H21	119.6
C30—N31—H31A	109.5	C20—C21—C22	120.77 (12)
C30—N31—H31B	109.5	C20—C21—H21	119.6
C30—N31—H31C	109.5	O3—C2—C4	117.66 (11)
O26—C25—O27	124.47 (11)	O1—C2—O3	123.96 (12)
O26—C25—C22	116.94 (10)	O1—C2—C4	118.37 (11)
O27—C25—C22	118.55 (11)	C19—C20—H20	119.8
C23—C24—H24	119.6	C21—C20—C19	120.39 (12)
C23—C24—C19	120.71 (11)	C21—C20—H20	119.8
C19—C24—H24	119.6	F14—C11—F13	107.21 (10)
C7—C8—H8	119.6	F14—C11—F12	106.88 (10)
C9—C8—H8	119.6	F14—C11—C10	110.86 (10)
C9—C8—C7	120.80 (11)	F13—C11—F12	106.08 (10)
C24—C23—H23	119.8	F13—C11—C10	113.79 (10)
C24—C23—C22	120.39 (11)	F12—C11—C10	111.63 (10)
C22—C23—H23	119.8	C7—C6—H6	119.9
C24—C19—C10	118.57 (11)	C5—C6—C7	120.29 (12)
C20—C19—C24	118.70 (11)	C5—C6—H6	119.9
C20—C19—C10	122.63 (11)	C4—C5—C6	121.31 (12)
C8—C7—C10	118.23 (10)	C4—C5—H5	119.3
C8—C7—C6	118.41 (11)	C6—C5—H5	119.3
C6—C7—C10	123.27 (11)	N28—C29—H29A	108.8
C8—C9—H9	119.6	N28—C29—H29B	108.8
C8—C9—C4	120.74 (11)	N28—C29—C30	113.89 (11)
C4—C9—H9	119.6	H29A—C29—H29B	107.7
C23—C22—C25	120.66 (11)	C30—C29—H29A	108.8
C23—C22—C21	118.97 (11)	C30—C29—H29B	108.8
C21—C22—C25	120.35 (11)	N31—C30—C29	114.34 (10)
C19—C10—C15	106.27 (10)	N31—C30—H30A	108.7
C19—C10—C11	111.49 (10)	N31—C30—H30B	108.7
C7—C10—C19	111.99 (9)	C29—C30—H30A	108.7
C7—C10—C15	112.66 (10)	C29—C30—H30B	108.7
C7—C10—C11	106.06 (10)	H30A—C30—H30B	107.6
C15—C10—C11	108.40 (10)		
			
O26—C25—C22—C23	157.85 (11)	C7—C10—C15—F16	−68.51 (13)
O26—C25—C22—C21	−20.38 (17)	C7—C10—C15—F17	171.16 (10)
O27—C25—C22—C23	−20.36 (17)	C7—C10—C11—F14	−72.89 (12)
O27—C25—C22—C21	161.41 (12)	C7—C10—C11—F13	166.18 (10)
N28—C29—C30—N31	72.72 (14)	C7—C10—C11—F12	46.15 (13)
C25—C22—C21—C20	177.17 (11)	C7—C6—C5—C4	−0.7 (2)
C24—C23—C22—C25	−176.28 (11)	C9—C8—C7—C10	−177.21 (11)
C24—C23—C22—C21	1.97 (18)	C9—C8—C7—C6	−0.52 (18)
C24—C19—C10—C7	−35.23 (15)	C9—C4—C2—O3	−1.95 (18)
C24—C19—C10—C15	88.18 (12)	C9—C4—C2—O1	176.99 (12)
C24—C19—C10—C11	−153.89 (11)	C9—C4—C5—C6	0.97 (19)
C24—C19—C20—C21	2.98 (19)	C22—C21—C20—C19	−1.4 (2)
C8—C7—C10—C19	−44.56 (14)	C10—C19—C20—C21	179.27 (11)
C8—C7—C10—C15	−164.30 (11)	C10—C7—C6—C5	177.01 (12)
C8—C7—C10—C11	77.27 (13)	C15—C10—C11—F14	165.90 (10)
C8—C7—C6—C5	0.50 (19)	C15—C10—C11—F13	44.96 (13)
C8—C9—C4—C2	179.77 (11)	C15—C10—C11—F12	−75.06 (13)
C8—C9—C4—C5	−0.99 (18)	C2—C4—C5—C6	−179.78 (12)
C23—C24—C19—C10	−178.54 (11)	C20—C19—C10—C7	148.47 (11)
C23—C24—C19—C20	−2.09 (18)	C20—C19—C10—C15	−88.12 (13)
C23—C22—C21—C20	−1.09 (19)	C20—C19—C10—C11	29.81 (16)
C19—C24—C23—C22	−0.38 (18)	C11—C10—C15—F18	169.15 (10)
C19—C10—C15—F18	−70.90 (12)	C11—C10—C15—F16	48.55 (13)
C19—C10—C15—F16	168.50 (10)	C11—C10—C15—F17	−71.78 (13)
C19—C10—C15—F17	48.17 (13)	C6—C7—C10—C19	138.92 (12)
C19—C10—C11—F14	49.27 (13)	C6—C7—C10—C15	19.19 (16)
C19—C10—C11—F13	−71.67 (13)	C6—C7—C10—C11	−99.25 (13)
C19—C10—C11—F12	168.30 (10)	C5—C4—C2—O3	178.82 (12)
C7—C8—C9—C4	0.78 (18)	C5—C4—C2—O1	−2.24 (18)
C7—C10—C15—F18	52.10 (13)		

### Ethane-1,2-diaminium 4,4'-(1,1,1,3,3,3-hexafluoropropane-2,2-diyl)dibenzoate monohydrate (2) Hydrogen-bond geometry (Å, º)

**Table:**

*D*—H···*A*	*D*—H	H···*A*	*D*···*A*	*D*—H···*A*
O32—H32*A*···O27^i^	0.85	1.92	2.7656 (14)	175
O32—H32*B*···O26^ii^	0.85	1.93	2.7731 (13)	170
N28—H28*A*···O1^iii^	0.91	1.87	2.7749 (14)	171
N28—H28*B*···O27	0.91	1.87	2.7609 (14)	165
N28—H28*C*···O3^iv^	0.91	2.00	2.8015 (14)	146
N31—H31*A*···O26	0.91	1.82	2.6933 (14)	160
N31—H31*B*···O32	0.91	1.91	2.7288 (14)	149
N31—H31*C*···O3^v^	0.91	1.91	2.7220 (14)	148

Symmetry codes: (i) *x*+1/2, −*y*+1/2, −*z*+1; (ii) −*x*+2, −*y*+1, −*z*+1; (iii) *x*+1/2, *y*, −*z*+1/2; (iv) −*x*+3/2, −*y*, *z*+1/2; (v) *x*, −*y*+1/2, *z*+1/2.
