# Hydrogen-bonding patterns in 2,2-bis­(4-methyl­phen­yl)hexa­fluoro­propane pyridinium and ethyl­enedi­ammonium salt crystals. Corrigendum

**DOI:** 10.1107/S2056989020013936

**Published:** 2020-10-31

**Authors:** Haruki Sugiyama

**Affiliations:** a Research and Education Center for Natural Sciences, Keio University, Hiyoshi, 4-1-1, Kohoku, Yokohama, Japan

## Abstract

In the paper by Sugiyama [*Acta Cryst.* (2020), E**76**, 742–746], there is an error in the chemical names of the title compounds.

## Abstract 2

Corrigendum to *Acta Cryst.* (2020), E**76**, 742–746.

The chemical names of the title compounds in the paper by Sugiyama (2012 ▸) are incorrect. The title should read ‘Hydro­gen-bonding patterns in pyridinium 2,2-bis­(4-carboxy­phen­yl)hexa­fluoro­propane and ethyl­enedi­ammonium salt crystals’, and ‘2,2-bis­(4-methyl­phen­yl)hexa­fluoro­propane’ should be ‘2,2-bis­(4-carboxy­phen­yl)hexa­fluoro­propane’ throughout. The systematic names of the compounds in the *Abstract* are correct.
